# Exosome origin determines cell targeting and the transfer of therapeutic nanoparticles towards target cells

**DOI:** 10.1186/s12951-018-0437-z

**Published:** 2019-01-25

**Authors:** María Sancho-Albero, Nuria Navascués, Gracia Mendoza, Víctor Sebastián, Manuel Arruebo, Pilar Martín-Duque, Jesús Santamaría

**Affiliations:** 1 Department of Chemical Engineering, Aragon Institute of Nanoscience (INA), University of Zaragoza, Campus Río Ebro-Edificio I+D, C/ Mariano Esquillor S/N, 50018 Zaragoza, Spain; 2 Networking Research Center on Bioengineering, Biomaterials and Nanomedicine, CIBER-BBN, 28029 Madrid, Spain; 3 Fundación Araid, 50009 Zaragoza, Spain; 4 Instituto Aragonés de Ciencias de la Salud (IACS/IIS Aragón), Centro de Investigación Biomédica de Aragón (CIBA), 50009 Zaragoza, Spain

**Keywords:** Exosomes, Gold nanoparticles, Selectivity, Fingerprint, NIR hyperthermia

## Abstract

**Background:**

Exosomes are considered key elements for communication between cells, but very little is known about the mechanisms and selectivity of the transference processes involving exosomes released from different cells.

**Results:**

In this study we have investigated the transfer of hollow gold nanoparticles (HGNs) between different cells when these HGNs were loaded within exosomes secreted by human placental mesenchymal stem cells (MSCs). These HGNs were successfully incorporated in the MSCs exosome biogenesis pathway and released as HGNs-loaded exosomes. Time-lapse microscopy and atomic emission spectroscopy allowed us to demonstrate the selective transfer of the secreted exosomes only to the cell type of origin when studying different cell types including cancer, metastatic, stem or immunological cells.

**Conclusions:**

In this study we demonstrate the selectivity of in vitro exosomal transfer between certain cell types and how this phenomenon can be exploited to develop new specific vectors for advanced therapies. Specifically, we show how this preferential uptake can be leveraged to selectively induce cell death by light-induced hyperthermia only in cells of the same type as those producing the corresponding loaded exosomes. We describe how the exosomes are preferentially transferred to some cell types but not to others, thus providing a better understanding to design selective therapies for different diseases.
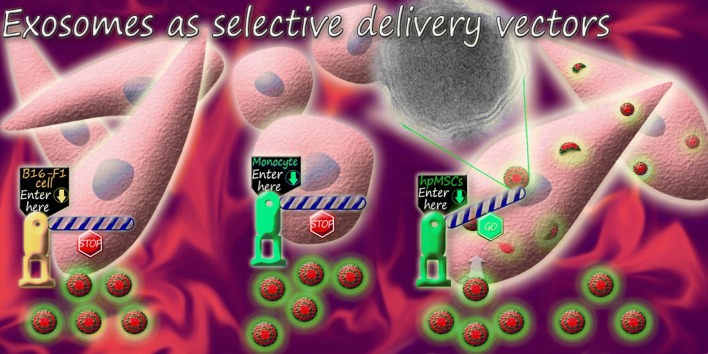

**Electronic supplementary material:**

The online version of this article (10.1186/s12951-018-0437-z) contains supplementary material, which is available to authorized users.

## Background

The body of an adult person contains around 37 billion cells that function coordinately [1]. To work as a whole entity many coordination mechanisms co-exist, using different factors as messengers. For example, the nervous system makes a strong use of communication by electrical impulses and the endocrine system is capable to send messages to distant areas mediated by hormones [2]. One of the most intensely studied at the moment concerns the exchange of genetic material and proteins mediated by exosomes or microvesicles secreted by the cells [3].

Many cell types present in the organism release vesicles of different nature, including apoptotic bodies, ectosomes, microvesicles and exosomes. Exosomes were known since 1981 when Trams and coworkers [4], defined exosomes as vesicles derived from the exfoliation of the plasmatic membrane, although the term “exosome” was coined in 1987 [5]. Early studies usually considered exosomes as “the garbage of the cells”, even though it was known that they contained genetic material (including mRNA, miRNA, DNA and proteins). Eventually, it was discovered that exosomes not only could serve as a mechanism to discharge unwanted material from cells, but also could form the basis of an efficient cell–cell communication mechanism [3, 6]. For instance, Valadi et al. showed that exosomal mRNA and micro RNA could be transferred to another cell being functional in this new localization [7].

Recent works dealing with the properties and functions of cell-derived exosomes suggest that they are involved in a variety of scenarios, including central nerve system diseases, myocardial ischemia/circulation damage, liver and kidney injury and the modulation of tumor hallmarks, inducing angiogenesis and metastasis [8]. Their role in cell physiology processes as immune-modulators and in regenerative processes in the body for the normal hemostasis maintenance has also been addressed [9]. Studying exosomal transfer between cells could provide key information on the evolution of different diseases. They also hold promise as a tool for allowing early diagnosis [10], since exosomes are present in most biological fluids (blood, urine, saliva, sperm, etc.) and therefore a variety of tests could be developed.

Another highly important characteristic of exosomes relates to their role as transference vectors of membrane receptors, functional proteins as growth factors or nucleic acids [11]. If this specific exosome-based transport could be controlled, it could be potentially used to transfer therapeutic elements (drugs, virus, nanoparticles, etc.). In fact, some investigations have already explored this path, harvesting exosomes and loading them with the desired therapeutics. Thus, Tian et al. used electroporation to load doxorubicin into exosomes derived from mouse immature dendritic cells, and then the drug-containing exosomes were targeted to tumors in vivo [12]. Similarly, Kim et al. used mild sonication to load paclitaxel into macrophage-produced exosomes and reported that the loaded exosomes could be used to treat carcinomas at lower drug doses than the ones used in conventional treatments [13]. However, electroporation and sonication can disrupt the exosomal membrane, and therefore other routes that exploit natural uptake mechanisms are preferred. Pascucci et al. were probably the first to show that an active drug (paclitaxel) could be selectively up taken by mesenchymal stem cells and then incorporated into the released exosomes in sufficient concentration to inhibit the growth of tumor cells in vitro [14]. Altanerova et al. reported the use of mesenchymal stem cells derived exosomes for magnetic hyperthermia applications in cancer therapy [15]. To this end, they added Venofer, an iron-sucrose complex, to the culture medium of mesenchymal stem cells and isolated the exosomes produced, which contained significant amounts of iron. This enabled them to induce magnetic hyperthermia by incubating tumoral cells with iron-containing exosomes.

The therapeutic potential of exosomes has prompted a number of exosome-based treatments now being explored in clinical trials for diverse pathologies, including sepsis, diabetes, cancer, wound healing or stroke. A recent search retrieved 78 current clinical trials involving exosomes as tools for diagnosis or therapy [16]. However, in spite of the fact that most of the proposed applications focus on the ability of exosomes to target specific cells or tissues, the understanding of their selective trafficking and communication mechanisms remains poor. In fact, the nature of selective fingerprinting of exosomes is still the subject of intense debate [17–19]. Some of the few works on this matter suggests that extracellular vesicles, including exosomes, can be incorporated by every cell type studied [20] but others show that exosomes can be used as drug carriers thanks to their ability to selectively target cells [21]. Rana et al. demonstrated that the exosomal tetraspanin-complexes define the acceptor ligand in cells, and consequently are involved in cell selection [21]. Toda et al. compared the internalization efficiency of U251- and astrocyte derived exosomes in U251 cells observing that the uptake of astrocyte derived exosomes was significantly lower than that obtained for U251-derived exosomes [22]. Hazan-Halevy et al. showed a cell-specific uptake of MCL exosomes by normal and by MCL derived patients’ B-lymphocytes [23].

Herein we have used relatively large entities (40 nm in diameter hollow gold nanoparticles (HGNs)) as tools to monitor the exosomal transference between cells. HGNs are especially suitable given the properties of gold (chemical inertness, biocompatibility and low toxicity), but also because HGNs present surface plasmon resonance (SPR) in the near-infrared region. As a consequence, when exposed to light with a suitable wavelength (ca. 700 to 1000 nm) HGNs behave as efficient transductors of light into heat, and can be used to destroy malignant cells by hyperthermia or to trigger other temperature-driven processes, including drug delivery and gene expression [24–26]. We have successfully incorporated HGNs into the MSC exosome biogenesis pathway. Using HGN-loaded exosomes, we were able to follow cell–cell communication, and in particular how the exosome cellular origin could affect HGNs transference between cells. We investigated whether HGN-loaded exosomes were similarly or differentially incorporated by cancer cells, metastatic cells, human placental mesenchymal stem cells (MSCs) and cells from the mononuclear phagocyte system (MPS). Our results indicate that the exosomal envelope is key to maintain the identity and compatibility of transference, providing a fingerprint that is responsible for the transfer selectivity. Finally, we have exploited this preferential transfer to selectively induce death by hyperthermia only in specific cells.

## Results

### Hollow gold nanoparticles characterization

**Fig. 1:**
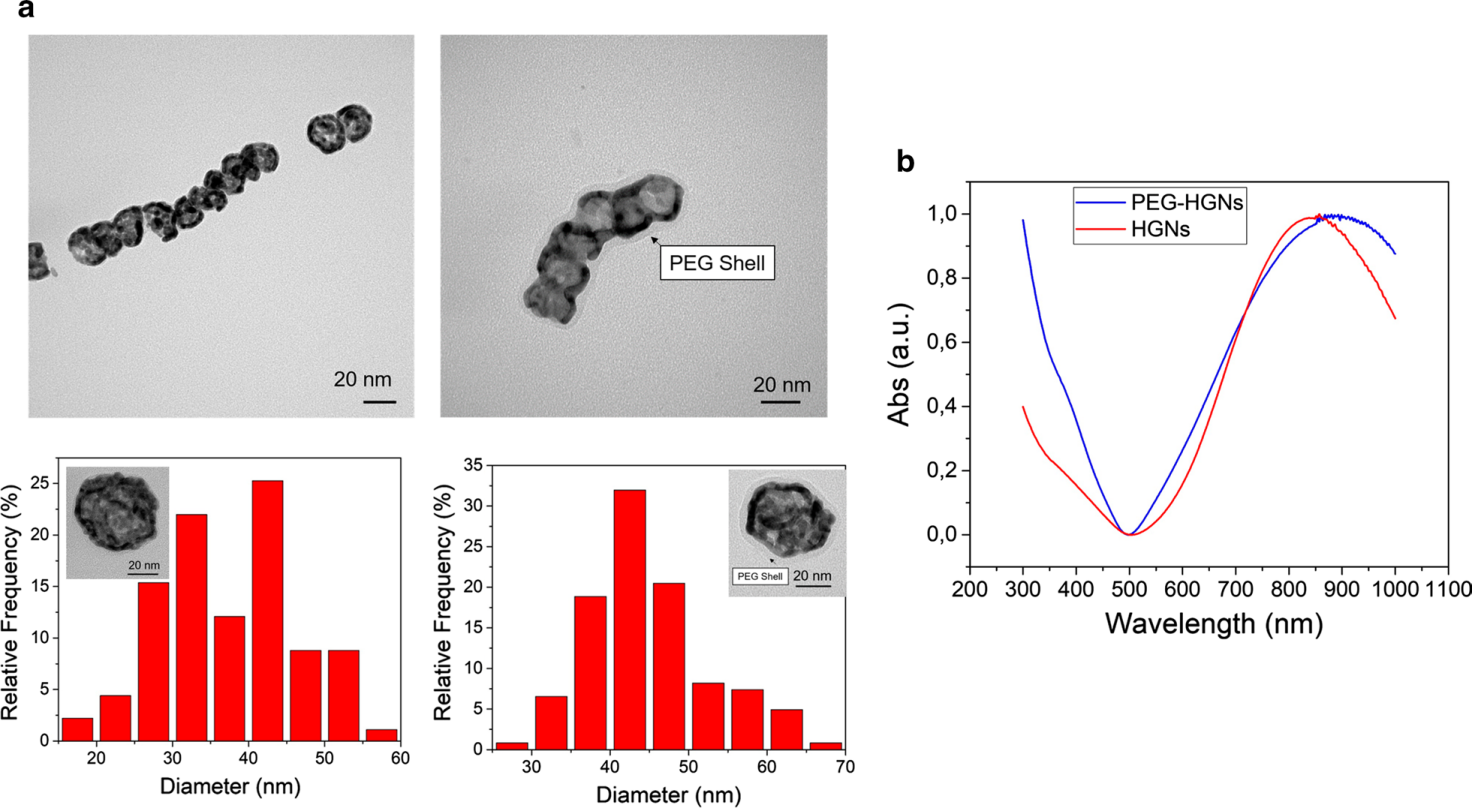
Characterization of HGNs and PEG-HGNs. **a** TEM images of HGNs and PEG-HGNs (above). PEG functionalization is clearly visible around the nanospheres by a negative staining to contrast the organic shell. Size distribution diagram of NPs obtained from TEM images (bottom). **b** UV–VIS absorption spectra for both types of nanoparticles. A maximum absorbance peak was observed at 850 nm, corresponding with the geometrical shape and size of these NPs

Transmission electron microscopy (TEM) images of the as-prepared nanoparticles (NPs) are shown in Fig. 1. The HGNs were hollow pseudo-spheres with a thick shell (Fig. 1a), and after polyethylene glycol (SH-PEG) functionalization, a uniform shell of almost 5 nm could be observed coating the HGNs. The mean diameter of HGNs obtained from the TEM images was 38.3 ± 8.3 nm, whereas the mean size of PEGylated NPs (PEG-HGNs) (determined by negative staining the TEM sample) was 44.9 ± 7.6 nm. Both HGNs and PEG-HGNs showed a characteristic localized surface plasmon resonance peak in the NIR region around 850 nm (Fig. 1b). A slight red shift was observed for the PEGylated NPs, possibly attributed to a different dielectric value in the interfacial double layer coating on the NPs [27]. Phase analysis light scattering measurements (not shown) showed that at pH = 7 the surface charge for the HGNs in water was − 15.35 ± 0.84 mV and compared to the one obtained for the PEG-HGNs (− 10.48 ± 0.35 mV) corroborated the efficient PEG coating on the particles surface.

The biological characterization of both HGNs and PEG-HGNs is thoroughly described in Additional file 1 section. The stability and the aggregation state of both HGNs and PEG-HGNs in cell culture media supplemented with 10% FBS was initially evaluated. Additional file 1: Figure S1A shows TEM images of both naked and PEGylated NPs after being in contact with cell culture media for 24 h (naked HGNs were significantly more aggregated than the PEGylated ones). In order to study the amount of protein adsorbed on the NPs, the bicinchoninic acid (BCA) assay was performed on both NP populations in cell culture media supplemented with 10% FBS. Additional file 1: Figure S1B evidences that in the case of HGNs the total protein amount adsorbed on their surfaces increased over time. On the contrary, the protein adsorption on PEGylated NPs was significantly lower compared to the amount adsorbed on naked HGNs in agreement with the well known steric hindrance attributed to PEG.

To corroborate this observation, the zeta potential of both NP-based dispersions in cell culture media was also measured, obtaining zeta potential values of − 3.64 ± 2.73 mV and − 8.50 ± 1.50 mV for HGNs and PEG-HGNs, respectively (Additional file 1: Figure S1C) which is indicative of a higher protein adsorption on the bare nanoparticles, shielding electric charge and giving zeta potential values closer to the isoelectrical point.

The dose-dependent viability of MSCs was evaluated under the presence of HGNs and PEG-HGNs by a metabolic assay. Additional file 1: Figure S2A reveals that 0.125 mg mL^−1^ was the limit for the subcytotoxic effect. Furthermore, we also confirmed that the presence of both types of NPs in the cell cultures did not produce significant changes on cell cycle phases at the subcytotoxic doses (Additional file 1: Figure S2B). Finally, the presence of the NPs inside MSCs was evaluated by confocal microscopy (Additional file 1: Figure S2C) and was indirectly quantified by MP-AES using the total amount of gold measured inside the cells (Additional file 1: Figure S2D). These results indicate that significantly more PEG-HGNs were localized inside cells compared to the amounts retrieved for HGNs, probably due to the higher stability and reduced agglomeration in the culture media provided by the PEG-surface functionalization. A better dispersion might increase the chances of nanoparticle internalization. Going deeper on HGNs uptake by MSCs, we also evaluated the preferential pathways that PEG-HGNs followed in the process of being incorporated into the target cells to verify if those strategies were compatible with the exosomal pathway. Additional file 1: Figure S3 reveals that not only clathrin-mediated endocytosis, but also other energy-dependent pathways were the main internalization mechanisms involved in the PEG-HGNs capture by MSCs, and therefore nanoparticles could be efficiently incorporated into the target cells.

### Secretion of empty and HGN-loaded exosomes by MSCs

**Fig. 2:**
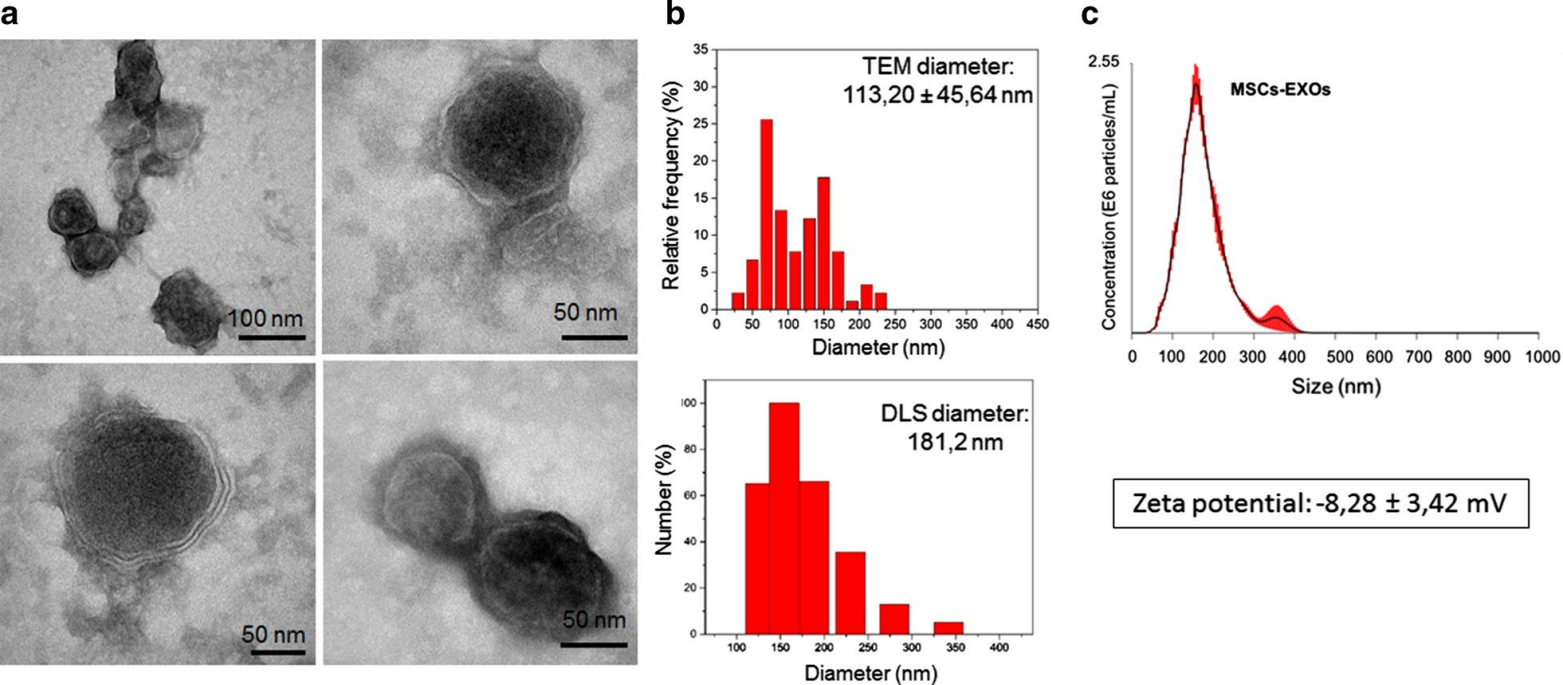
Characterization of MSCs-EXOs produced in the absence of HGNs. **a** Representative TEM images of an exosome sample isolated from MSCs cells supernatants. In the bottom image the double lipid membrane is clearly visible. **b** Size distributions derived from TEM images (above) and from DLS (bottom). **c** NTA results of MSCs_EXOs in PBS

Before attempting to encapsulate HGNs within exosomes, we characterized MSCs derived exosomes (MSCs-EXOs) by physico-chemical and biological techniques. TEM images of MSCs-EXOs isolated from culture supernatants by successive ultracentrifugation steps are shown in Fig. 2. A spherical shape and a characteristic lipidic bi-layer membrane were observed in most of them. The size distribution histograms of exosomes obtained from TEM images, gave an average diameter of 113.2 ± 45.6 nm. Dynamic light scattering (DLS) and nanoparticle tracking analysis (NTA) measurements gave a mean particle diameter around 180 nm in PBS. The measured surface charged of exosomes at pH 7 was − 8.28 ± 3.42 mV likely due to the negatively charged phospholipids and proteins present on the exosomal membrane.

### Incorporation of HGNs into MSCs-derived exosomes

**Fig. 3:**
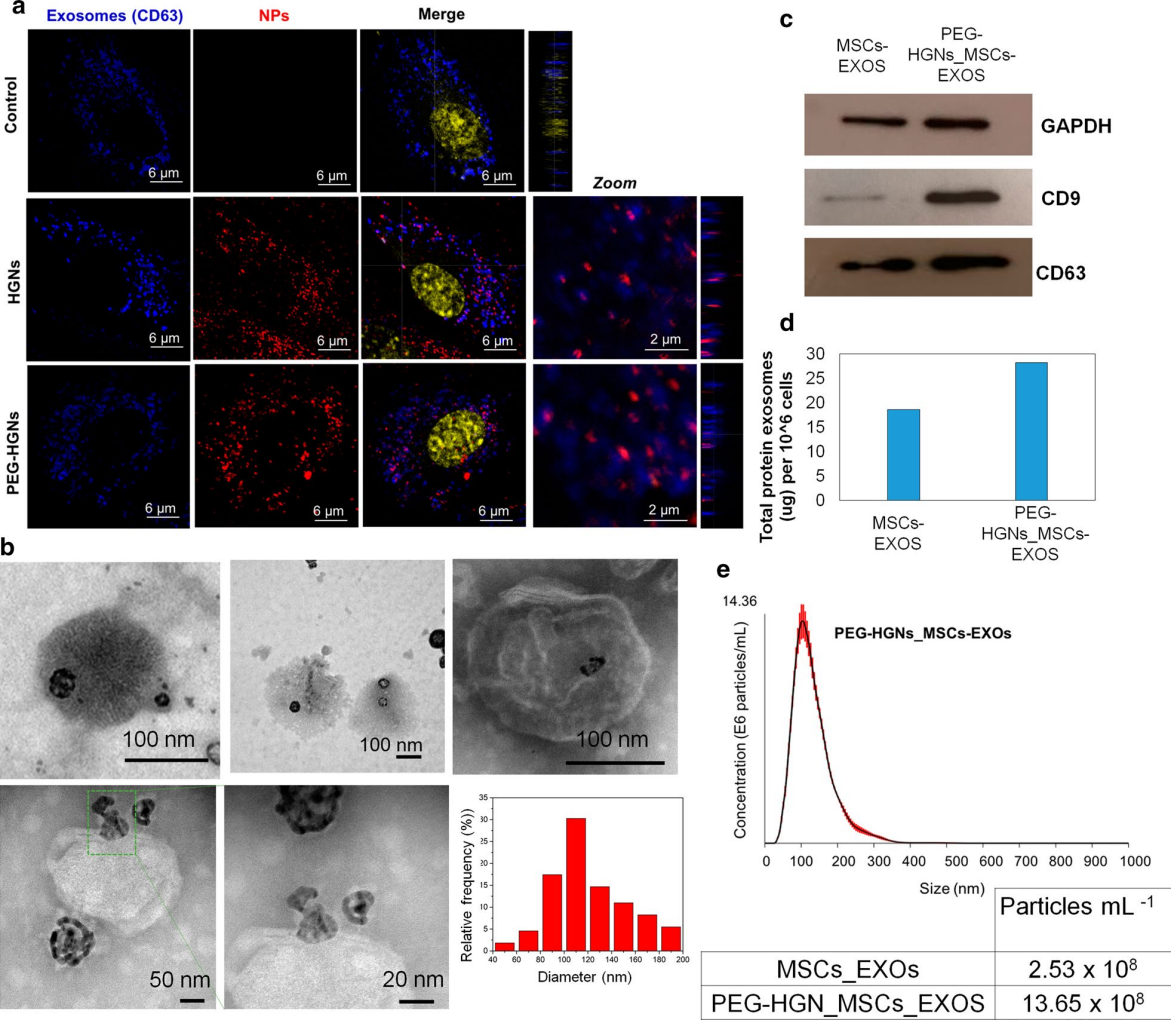
Location of PEG-HGNs in exosomes derived from MSCs. **a** Intracellular localization of HGNs and PEG-HGNs in MSCs by confocal microscopy. Most of the HGNs and PEG-HGNs co-localized with exosomes stained with CD63 antibody. There was also a smaller fraction of NPs inside the cytoplasm without co-localization with exosomes. Yellow represents nuclei stained by DAPI, blue are the exosomes labeled with CD63-488 antibody, and NPs were directly visualized by reflection. **b** TEM images of exosomes purified (ultracentrifugation) from MSCs incubated with PEG-HGNs for 48 h and size distribution histogram. Most exosomes were loaded with NPs. **c** Western Blot of MSCs derived exosomes with or without PEG-HGNs. **d** Total protein exosomes quantification by BCA assay when cells were treated under the presence or absence of PEG-HGNs. **e** NTA measurements of exosomes sizes and concentrations

We have used confocal microscopy to monitor the association of HGNs with exosomes. To this end, exosomes were labeled with a specific CD63-Alexa488 antibody (blue) the cell nuclei with Draq-5 (yellow) and the HGNs agglomerates were directly observed by reflection (red) (Fig. 3a). Orthogonal projections from confocal laser-scanning microscopy analysis revealed high purple fluorescence pixels on the MSCs cytoplasm, i.e., the merging of the blue and red fluorescence, which corresponds with aggregates of NPs and exosomes or late endosomes co-localization, indicating that multivesicular bodies within the cell already contain HGN-loaded exosomes. After these were excreted, the HGN-loaded exosomes could be easily recovered by ultracentrifugation and subjected to TEM analysis. TEM images of purified exosomes from MSCs culture supernatants after incubation with PEG-HGNs showed that most exosomes were loaded with at least one nanoparticle, confirming the high yield of nanoparticle loading into exosomes (Fig. 3b). The diameter distribution histogram of PEG-HGNs_MSCs-EXOs obtained from TEM images revealed average sizes of 126.9 ± 39.2 nm. These values confirmed that the presence of the NPs inside the exosomes did not affect their endogenous diameter. Furthermore, the size distributions obtained by NTA for control exosomes and for PEG-HGNs_MSCs-EXOs (Figs. 2c, 3e, respectively) were also similar. The measured surface charged of PEG-HGNs_MSCs-EXOs at pH 7 was − 10.26 ± 0.17 mV, confirming that the presence of the PEG-HGNs did not alter the negatively charged phospholipids and proteins present on the exosomal membrane. Western Blot analysis of isolated vesicles showed expression of the exosome-associate proteins CD9 and CD63 [signal at 21 and 53 kDa respectively, were clearly observed (Fig. 3c)]. Comparing total protein amounts obtained by the BCA (Fig. 3d) and exosomes concentration measured by NTA (Fig. 3e), higher amounts of exosomes were significantly secreted when cells were treated with NPs compared to the amounts measured for control samples (untreated cells).

### Exosomes as specific vectors of PEG-HGNs between different cell lines

Given the way nanoparticle-loaded exosomes are generated, we postulated that exosomes derived from a cell line are fingerprinted with hallmarks of that cell type and therefore would be preferentially up-taken by the same cell line, even under co-culture conditions with other cell lines. To verify if exosomes from a cell line were specifically captured by the same cell line we developed a co-culture of stem cells and monocytes under optimized conditions for their simultaneous growth. To facilitate identification, both types of cells were labeled with PKH67 (green) and PKH26 (red) dyes, respectively (Additional file 1: Figure S4). Once co-cultures were optimized, the exchange of material between cells (i.e., the intercellular trafficking among MSCs and between MSCs and monocytes) could be easily followed by time-lapse microscopy thanks to the HGNs loaded inside the exosomes.

**Fig. 4:**
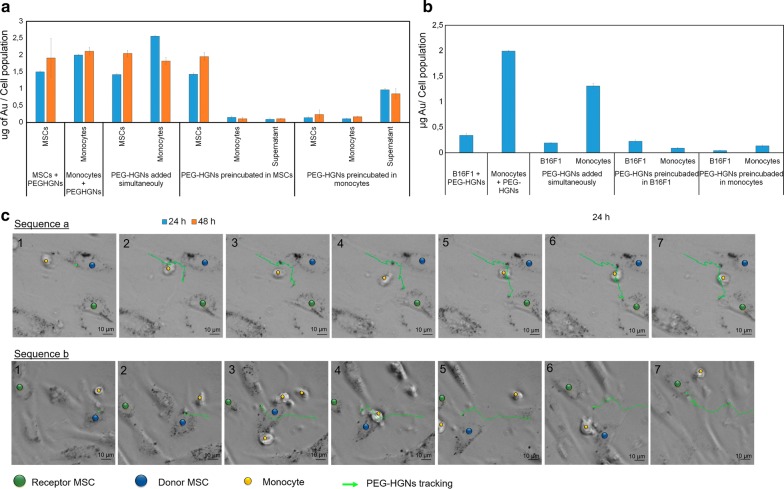
NPs trafficking between different cell lines. **a** MP-AES assay to quantify nanoparticle distribution (gold content) between monocytes and MSCs when the PEG-HGNs were incubated simultaneously with either or with both cell types, when nanoparticles were previously incubated with MSCs, or when they were pre-incubated with monocytes. **b** A similar MP-AES series of experiments of gold distribution was carried out between tumoral (B16-F1) cells and monocytes. **c** Time-lapse images corresponding to the transfer of PEG-HGNs between MSCs (marked with blue and green dots). The frames have been selected because in both cases there were monocytes close by that did not take the passing by nanoparticles. Time-lapse videos showing these and other examples of selective transfer are available as Additional files 2 (PEG-HGNs added simultaneously) and 3 (PEG-HGNs transference among MSCs)

We performed time-lapse microscopy of MSCs and monocytes cultured separately (control) and co-cultured for 3 days to observe nanoparticle transference in real time. We also used MP-AES to determine the Au content of the different cell populations and supernatants in order to follow overall internalization of PEG-HGNs. Our results (Fig. 4) show that, when fresh nanoparticles (i.e. nanoparticles that had not previously been incubated with any type of cell) were added either to each cell type individually or to co-cultures containing simultaneously MSCs and monocytes, they were significantly up-taken by both cell types (between 1 and 2 μg of gold/cell population). In contrast, when PEG-HGNs were pre-incubated in MSCs and then PEG-HGNs loaded exosomes were put in contact with monocytes, we observed that nanoparticles transference occurs preferentially between MSCs and only a minor fraction of PEG-HGNs was captured by monocytes both at 24 h and 48 h (0.09 μg of gold/cell population at both times). The symmetric experiment was carried out by pre-incubating PEG-HGNs in monocytes and co-culturing them with MSCs. In this case, the amount of gold nanoparticles internalized in MSCs was remarkably small, in spite of the fact that the supernatant concentration was high (0.9 μg of gold/cell population), i.e. there was a large concentration of nanoparticles available. It is reported that when monocytic cells recognize nanoparticles in the media, as efficient scavengers, they firstly internalize NPs and trap them in the endosomal-exosomal pathway releasing those materials again to the media [28]. Figure 4 shows that the majority of gold released to the media by monocytes remained in the media instead of being uptaken by MSCs, thus confirming the preservation of the self-signature of monocytes.

Moreover, the same trend was also observed in Additional file 1: Figure S5. Herein, we employed MP-AES to determine the specific capturing of previously purified PEG-HGNs loaded exosomes of different cellular origin. When MSCs derived exosomes loaded with PEG-HGNs (PEG-HGNs_MSCs-EXOs) were added to a co-culture of MSCs and monocytes, they were preferentially taken by MSCs (0.12 and 0.17 μg of gold/cell population at 24 h and 48 h, respectively) and only a minor fraction of PEG-HGNs was internalized into monocytes (0.015 μg of gold/cell population). In the symmetric experiment, (i.e. PEG-HGNs were pre-incubated with monocytes and then the exosomes produced were released into the co-culture of MSCs and monocytes) the amount of gold nanoparticles internalized in MSCs was remarkably small, in spite of the fact that the supernatant concentration was high (0.14 μg of gold/cell population). While the Au analysis results agree with the selective uptake of PEG-HGNs-loaded exosomes according to their cellular origin, Fig. 4 and Additional file 1: Figure S5 also seem to indicate that, to a lesser extent, some type of non-selective internalization could also be taking place, since a small fraction of Au was observed to be internalized by non-related cells. However, this can be explained as a result of the uptake of free Au nanoparticles that either were present originally in the medium or were excreted through a non-exosomal pathway and therefore lack the cell-specific exosomal coating. These free HGNs were clearly present along with exosome-enclosed HGNs in the culture medium (see Fig. 3b).

To validate the conclusions reached with other cell types, the same study was carried out using monocytes and tumoral cells (B16-F1 cells) instead of MSCs. The results (Fig. 4) show that for separate cultures of each cell, the uptake of PEG-HGNs by monocytes was significantly (ca. 5 times) higher than that obtained for the tumoral cells. When cultured together, a similar uptake ratio is observed for both types of cells. However, when the gold nanoparticles were pre-incubated in B16-F1 cells and transferred to the co-culture with monocytes, only a small amount of PEG-HGNs was captured by monocytes (0.089 μg of gold/cell population). On the contrary, when PEG-HGNs were pre-incubated with monocytes and the resulting HGN-containing exosomes were put in contact with the co-culture of monocytes and B16-F1 cells, only a small fraction was captured by B16-F1 cells (0.042 μg of gold/cell population).

Time-lapse microscopy was used to obtain a direct confirmation of the conclusions obtained from MP-AES analysis regarding the selective uptake of PEG-HGNs according to their origin. To this end, MSCs were incubated with PEG-HGNs and then the MSCs containing internalized nanoparticles were co-cultured with monocytes. As can be seen in the frames presented in Fig. 4c and d, and in the movies included as Additional files 2 and 3, a high amount of nanoparticles were present inside the MSCs, and those were very active in transferring nanoparticles between cells. However, tracking of the excreted material shows that the nanoparticles released by a donor MSC were mostly captured by another MSC and not by monocytes present in the proximity, even though monocytes were close by and often in the path of the released material. Therefore, a specific signature of the exosomes released from a cell type is conserved and used as recognition moiety for the same cell type.

### Selective death by hyperthermia mediated using PEG-HGNs-loaded exosomes

As a proof of concept of the possibility of inducing selective cell death using the high intrinsic selectivity of exosome-mediated transport we analyzed the in vitro photothermal effect of HGN-loaded exosomes originated from MSCs on separate cultures containing MSCs, melanoma cells (B16-F1 and B16-F10) and monocytes. To this end, MSCs were incubated with PEG-HGNs (0.1 mg mL^−1^) and the resulting HGN-containing exosomes were harvested and purified. Then cultures with the above cell lines were exposed to the loaded exosomes for 24 h (the PEG-HGNs_MSCs-EXOs concentration added to each well, was estimated as the ratio between the amount of donor cells from which those exosomes were derived (MSCs) and the number of treated cells). After washing, cell cultures were irradiated with a NIR laser, and viability/toxicity was evaluated by flow cytometry as well as by direct live/dead staining.

**Fig. 5:**
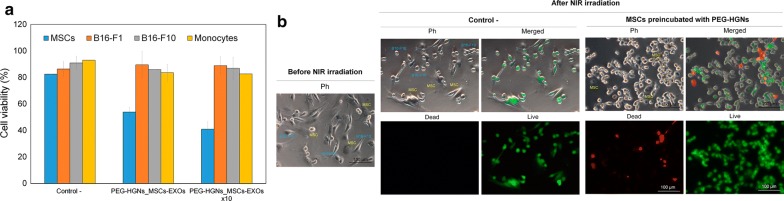
Photothermal effects mediated by MSCs-derived exosomes containing plasmonic HGNs. **a** Quantification of cell death when MSCs, monocytes, B16-F1 and B16-F10 cell lines cells were treated with PEG-HGNs-containing exosomes derived from MSCs. **b** Photothermal effect in a co-culture of PEG-HGN containing MSCs and B16-F1 cells during 48 h before subjecting the culture to NIR irradiation

In Fig. 5a, it is possible to observe that NIR laser irradiation did not significantly reduce cell viability on B16-F1 cells, B16-F10 cells and monocytes that had been treated with PEG-HGNs containing exosomes derived from MSCs, i.e., they present the same viability as the control samples, (cells subjected to NIR irradiation in the absence of HGNs loaded exosomes). On the contrary, a reduction in viability of almost 50% was observed for irradiated MSCs after treatment with HGN-loaded exosomes derived from MSCs. Very similar results were obtained using a fluorescence inverted microscope to quantify live/dead cells after exposure to MSC-derived exosomes followed by NIR irradiation (Additional file 1: Figure S6). These results again confirm the high specificity of exosome uptake depending on the exosomes origin. HGNs_MSCs-EXOs produced by seeded cells to treated cells, a decrease in the viability of approximately 70% was measured for irradiated MSCs demonstrating a dose-dependent process. In contrast, the viability of B16-F1 cells, B16-F10 cells and monocytes again was unaffected.

Finally, a further study was carried out to confirm selective exchange in the dynamic environment of a co-culture system. To this end, MSCs previously loaded with PEG-HGNs were co-cultured with B16-F1 cells during 48 h, a period sufficiently long to have multiple excretions/uptakes of nanoparticles by MSCs. Figure 5b shows that, after laser irradiation, only MSCs were dead while B16-F1 were still alive. This selective photothermal effect confirmed the lack of exosomal exchange between B16-F1 and MSCs also under co-culture conditions and this fact is consistent with our hypothesis of exosome fingerprinting according to the cellular origin.

## Discussion

Thanks to the intrinsic size of the nanomaterials and considering the size of the cellular components and that most of the biological processes occur at the nanometer scale, a novel and promising field has been opened. Thus, research on exosomes and NPs combination has gained attraction over the last decade [29–31]. Several works have reported the unique propierties of HGNs as efficient transductors of light into heat, being used to destroy malignant cells by hyperthermia or to trigger other temperature-driven processes, such as drug delivery and gene expression [24–26]. Although exosomes are considered key elements for communication between cells, few studies are reported about the mechanisms and selectivity of the transference processes involving exosomes released from different cells [17, 18]. Herein we study the selectivity of in vitro exosomal transfer between certain cell types and how this phenomenon can be exploited to develop new specific vectors for the transfer of relatively large entities (40 nm in diameter HGNs) between different cells when these NPs were loaded within exosomes secreted by human MSCs.

As was previously reported by other authors, the morphology and the plasmonic response of the HGNs and PEG-HGNs correspond with the ones described in the literature [32]. Amongst the characteristics of those NPs, the presence of SH-PEG coating is usually employed to provide a stealth cover to retard detection and removal by MPS macrophages by reducing the protein corona formation around HGNs. Also, both NPs dispersions have a maximum resonance peak at 808 nm, in which the absorption of incident light is minimized in biological fluids because of the reduced light absorption by water and chromophores [33].

Regarding the cytotoxicity test and the cellular cycle analysis, our results indicate that the presence of both types of NPs did not produce significant effects on cell viability and on cell cycle distribution at the studied dose. These results are in agreement with previous studies in our laboratory describing the lack of toxicity of HGNs and especially PEG-HGNs in tumoral cells, bone marrow derived stem cells and fibroblasts [34]. Moreover, the amount of particle uptake by MSCs after 24 h of incubation are within the wide interval found in previous internalization studies with different cells and particles (values reported ranged from 10^3^ to 10^7^ particles per cell) [35].

Both the size distribution histograms obtained and the DLS measurements obtained for MSCs-EXOs were in agreement with previously reported diameters of MSCs-EXOs [36]. The measured surface charged of exosomes was attributed to the negatively charged phospholipids and proteins present on the exosomal membrane. This value was in accordance with previous literature [37].

In previous reports, it has been demonstrated that EVs enter cells by a variety of endocytic pathways, including clathrin-dependent endocytosis, and clathrin-independent pathways such as caveolin-mediated uptake, macropinocytosis, phagocytosis, and lipid raft-mediated internalization [38]. Phagocytosis, clathrin-dependent endocytosis, caveolae-mediated endocytosis, endocytosis toward receptors and pinocytosis are reported as the main cellular uptake pathways of NPs [39]. However, the mechanism may change depending on particle size, surface chemistry and shape [40]. Also, even for the same type of NPs, the internalization mechanisms may be different for different cell lines [41]. As noted above, our results indicate HGNs and PEG-HGNs are internalized by MSCs following energy-dependent and clathrin-mediated endocytosis processes. Furthermore, nanoparticles are localized within early endosomes after 5 h of incubation. Soon afterwards PEG-HGNs can be observed within multi-vesicular bodies (MVB), and co-localize with exosomes labeled with a specific antibody (CD63). As previously shown, an intense production of exosomes loaded with PEG-HGNs was observed by CD9 and CD63 expression by the BCA analysis and by the NTA results. These data indicate that the presence of PEG-HGNs within the cells did not significantly affect the protein content of exosomes. In fact, the intensity of the CD9 and CD63 bands was higher compared to the controls when MSCs were in contact with PEG-HGNs. These results were in accordance with the BCA and NTA analyses, which indicated that in presence of PEG-HGNs, MSCs exosomal protein secretion was enhanced compared to the control MSCs without added HGNs (Fig. 3d). Thus, it appears that when MSCs were incubated with HGNs, they release exosomes more intensely. This characteristic seems to agree well with earlier reports on the enhanced cell processes of MSCs exposed to PEG-HGNs [34].

Thereby, we have taken advantage of the intrinsic exosome biogenesis process to obtain exosomes loaded with gold NPs (Additional file 1: Scheme S1) by adding PEG-HGNs to the cell culture medium of MSCs. These vesicles could be recovered easily, since HGN-containing exosomes precipitated readily by ultracentrifugation. This loading process avoids harsh treatments such as membrane rupture by electroporation or chemical bonding on the membrane wall and is likely to preserve better the natural characteristics of the produced exosomes. Few studies have attempted the encapsulation of preformed nanoparticles within exosomes, generally using exogenous methods to achieve encapsulation. For instance, Hood et al. [42], loaded superparamagnetic iron oxide nanoparticles (SPIONs) inside exosomes by electroporation, obtaining B16-F10 derived exosomes loaded with 5 nm iron nanoparticles. They proposed the use of semi-synthetic exosomes for diagnosis or therapeutic applications. Furthermore, Hu et al. also used electroporation to load SPIONs into B16-F10 derived exosomes for imaging and tracking by MRI in vitro and in vivo [29]. Also, several works have described the grafting of gold nanoparticles to the exosome surface via affinity agents such as lectins and antibodies [43]. Roma-Rodrigues et al. [44] used gold nanoparticles functionalized with thiolated oligonucleotides for selective silencing of RAB27A gene (essential gene for the biogenesis and processing of exosomes) with a consequent decrease of exosomes release. Other authors have employed more gentle or natural methods to load nanoparticles into exosomes. Thus, Alhasan et al. [30] loaded gold nanoparticle-nucleic acid constructs within exosomes by incubating them with prostate cancer cells and allowing endocytosis. A small fraction (< 1%) of the gold was then released inside exosomes, and could be recovered and re-introduced by exposing the cells to the recovered exosomes. In a different approach, Betzer et al. used the GLT-1 transporter to induce loading of glucose-coated gold nanoparticles into exosomes as labels for in vivo neuroimaging [31].

Once nanoparticle-loaded exosomes were generated, we evaluated if those exosomes were fingerprinted and therefore would be preferentially captured by the same cell line, even under co-culture conditions with other cell lines. Both MP-AES evaluation and time-lapse image sequences demonstrate that the nanoparticles released by a donor MSCs were rather up-taken by another MSCs and not by monocytes present in the proximity. Therefore a specific signature of the exosomes released from a cell type is conserved and used as recognition moiety for the same cell type. Although there are few works postulating that exosomes can be incorporated by every cell type [20], our results are in agreement with previous works suggesting that exosomes have the ability to target specific cells and to serve as selective vehicles [21–23].

The highly selective transfer mechanism mediated by exosomes opens the possibility to use these vectors for targeted therapy. Nanoparticles are particularly attractive in a scenario of therapeutic nanoparticle delivery, since their loading within exosomes would help to avoid in vivo recognition by the MPS. As a proof of concept of the possibility of inducing selective cell death using the high intrinsic selectivity of exosome-mediated transport we analyzed the in vitro photothermal effect of HGN-loaded exosomes originated from MSCs on separate cultures containing MSCs, melanoma cells (B16-F1 and B16-F10) and monocytes. Our results again confirm the high specificity of exosome uptake depending on the exosomes origin (Fig. 5). The selective transference of exosomes loaded with NPs among the same cell line was also confirmed in the dynamic environment of a co-culture system observing that only MSCs were dead while B16-F1 were still alive after NIR irradiation when MSCs were loaded with PEG-HGNs and then were co-cultured with B16-F1. This selective photothermal effect clearly demonstrates the absence of exosomal exchange between tumoral and MSCs also under co-culture conditions, and is consistent with our hypothesis of exosome fingerprinting according to the cellular origin. To our knowledge, this is the first work in which the specific transfer of exosomes is employed for selectively applied optical hyperthermia therapies.

## Conclusion

The encapsulation of PEG-HGNs in exosomes using the intrinsic biogenesis pathways of MSCs allowed us to confirm the high specificity of exosome-mediated cell exchanges, according to the cell origin. A wide study has been carried out with cells of different lineage, including stem cells, immune system cells, and tumor and metastatic cell lines. As a proof of concept of exosome-mediated selective transfer of therapeutic material into the targeted cells, for the first time our study demonstrates the specific in vitro optical hyperthermia therapy based on exosome delivery of NIR-responding plasmonic nanoparticles. Finally, we can conclude that considering the cell of origin and the compatibility of the target cells are of great importance when designing exosome-based therapies.

## Methods

### Synthesis and characterization of HGNs

HGNs synthesis was carried out according to the protocol developed by Preciado-Flores et al. [32] with slight modifications previously reported by Encabo-Berzosa et al. [34]. The complete synthesis protocol is described in Additional file 1 and the physico-chemical characterization of the nanoparticles was performed as previously reported [34].

### Biological evaluation of NPs

To evaluate the stability of HGNs and PEG-HGNs in cell culture media at 0.125 mg mL^−1^, the BCA assay (ThermoFisher, USA) was performed as mentioned before to evaluate the total amount of proteins adsorbed on them. Moreover, their aggregation state was assessed by TEM and by Zeta potential analysis. Blue Cell viability assay ^®^ (Abnova, USA) was employed to determine cell viability of MSCs under the effect of HGNs and PEG-HGNs [34]. To analyze the effects of HGNs and PEG-HGNs in cell cycle and in DNA damage, the distribution of cell cycle phases after NPs treatment was assessed by flow cytometry [34].

### Cellular uptake of NPs by MSCs

#### Confocal microscopy, flow cytometry and MP-AES

The cellular uptake and the trafficking of nanoparticles in MSCs were evaluated by confocal microscopy and the amount of gold inside MSCs was quantified by MP-AES using a quadrupole ICP mass spectrometer (4100 MP-AES, Agilent Technologies, USA) (see Additional file 1).

#### Identification of the cellular pathway

To study the specific internalization mechanism of NPs in MSCs, different uptake pathways were blocked with a variety of chemical inhibitors previously reported [45]. The presence or absence of HGNs and PEG-HGNs in MSCs under the effect of the different inhibitors was detected by Z-stack orthogonal projections obtained from confocal microscopy (Spectral Confocal Microscope Leica TCA SP2) as mentioned above. For more details see Additional file 1.

### Exosome isolation and characterization

MSCs-derived exosomes (MSCs-EXOs) were isolated following a protocol based on successive ultracentrifugation cycles from cell culture supernatants of MSCs throughly described in Additional file 1.

### Gold nanoparticles-loaded exosomes

In order to obtain PEG-HGNs_MSCs-EXOs, cells were cultured with ultracen medium enriched with PEG-HGNs at the subcytotoxic dose (0.125 mg mL^−1^) for 24 h. Then, the culture media was replaced by exosomes free medium for 48 h and excreted PEG-HGNs_MSCs-EXOs were purified from the supernatant as mentioned above. The presence of PEG-HGNs in exosomes was evaluated by confocal microscopy and by TEM as detailed in Additional file 1. Protein expression (GAPDH, CD9 and CD63) was evaluated by Western Blot analysis charging exosomes secreted by the same number of cells in order to be able to compare the protein expression when cells were treated or not with nanoparticles. A Pierce BCA protein assay was also performed as mentioned above to estimate the protein content in the exosomes sample secreted by MSCs when cells were treated or not with NPs. Furthermore NTA anlysis was carried out to evaluate the diameter and the number of particles per mL of control exosomes or PEG-HGNs_MSCs-EXOs. Exosomes measured by NTA were purified from the same number of cells under the presence or absence of PEG-HGNs.

### Exosomes as selective vectors of PEG-HGNs uptake between different cell lines

#### Optimization of co-culture conditions by flow cytometry and fluorescence microscopy

The evaluation of PEG-HGNs transference through exosomes between monocytes and MSCs was assessed to study the potential use of exosomes as nanoparticle specific vectors between different cell lines. In order to optimize the co-culture conditions, specific cell surface markers of monocytes and MSCs (CD19, CD14, CD34, CD45, CD73, CD90, CD10 and, HLA-DR) were evaluated by flow cytometry (FACSAria BD cytometer, BD Bioscience) when cells were cultured separately or together (see Additional file 1).

#### PEG-HGNs distribution between monocytes and MSCs

The in vitro distribution of of PEG-HGNs_MSCs-EXOs and monocytes derived exosomes loaded with nanoparticles (PEG-HGNs_monocyteexos), between both cell lines was visualized at real time by time-lapse microscopy with a 20× objective (Leica AF6000 LX, Germany). On the one hand, MSCs and monocytes were cultured separately as control condition in an IBIDI μ-Slide 8 well at a density of 1 × 10^5^ cells per well. On the other hand, 5 × 10^4^ monocytes were co-cultured together with 5 × 10^4^ MSCs in the same well for the co-culture samples. In these co-culture samples, two different experiments were carried out. First, the uptake and the in vitro distribution of PEG-HGNs_MSCs-EXOs and PEG-HGNs_monocyte-EXOs between both cell lines were evaluated when nanoparticles were added (0.125 mg mL^−1^) simultaneously to a cell co-cultured during 72 h. Secondly, PEG-HGNs were previously incubated with MSCs for 24 h and after that, 5 × 10^4^ MSCs containing nanoparticles were cultured with 5 × 10^4^ monocytes in an IBIDI μ-Slide 8 well as mentioned above. Samples were directly visualized by time-lapse microscopy to evaluate the specific transference of PEG-HGNs in exosomes between MSCs and monocytes after 24, 48 and 72 h of culture under the different conditions. Frames were taken every 15 min for 3 days at 37 °C and under normoxic conditions in DIC mode (Leica AF6000 LX, Germany). Finally, an image sequence was created and the specific cellular transference of PEG-HGNs_MSCs-EXOs between cells was tracked using ImageJ software. We created the track segments by linking consecutive frames, detecting the transference of nanoparticles between MSCs when a monocyte was present.

The selective uptake of exosomes loaded with PEG-HGNs between monocytes and MSCs under the different conditions assayed was also quantified by MP-AES, analyzing the gold content present in each cell type as well as that present in the supernatant. Again, cells were cultured separately or both together. To do that, they were seeded onto 6-well plates at a density of 2.5 × 10^5^ cells per well. Furthermore, 1.25 × 10^5^ monocytes were co-cultured with 1.25 × 10^5^ MSCs in the same well. After 24 h of maintenance, PEG-HGNs were added (0.125 mg mL^−1^) and incubated for 24 and 48 h. As mentioned above, nanoparticles were also pre-incubated with MSCs for 24 h and then 1.25 × 10^5^ MSCs loaded with PEG-HGNs were put in contact with 1.25 × 10^5^ monocytes for 24 and 48 h. The experiment was also carried out in the same way, but nanoparticles were also pre-incubated with monocytes instead of with stem cells. Once the cellular pellets were collected, they were digested with 10% Aqua regia (HNO_3_ + 3HCl) in dH2O (1.5 mL). Gold content of supernatants was also measured. Digestion was performed at room temperature for 1 h. Total amounts of gold derived from HGNs and PEG-HGNs was determined by MP-AES using a quadrupole mass spectrometer (4100 MP-AES, Agilent Technologies, USA). Calibrations were carried out using Au standards in 10% regia water ranging from 0 to 10 ppm.

A similar experiment was carried out by firstly purifying PEG-HGNs loaded exosomes from MSCs and from monocytes as mentioned above. Later, they were added to a co-cultured of MSCs and monocytes for 24 and 48 h in MW6 as it has been describe. Once cellular pellets were collected, their gold content was evaluated my MP-AES as explained before.

### Cell irradiation. Selective photothermal effect of PEG-HGNs_MSCs-EXOs

To study the laser photothermal effect in MSCs, melanoma cells (B16-F1 and B16-F10) and monocytes they were seeded (1.5 × 10^4^) onto a 48-well plate for 24 h after the incubation with PEG-HGNs_MSCs-EXOs. The PEG-HGNs_MSCs-EXOs concentration added to each well, was estimated as the ratio between the amount of donor cells from which those exosomes were derived (MSCs) and the number of treated cells. Ten times more of the amount of PEG-HGNs_MSCs-EXOs produced by seeded cells was also added to treated cells. To reduce any possible heating potentially produce by non-internalized exosomes present in the medium, cells were washed twice with PBS (1500 rpm, 5 min). All cellular types were then irradiated during 30 min with a NIR laser (808 nm) at 2.5 W cm^−2^ of irradiance. After that, cells were incubated with the LIVE/DEAD kit (Thermo Fisher Scientific, USA) following manufacturer instructions. Briefly, a solution containing 2 μM calcein AM and 4 μM ethidium homodimer-1 in PBS was prepared and added to the cells for 30 min at 37 °C. The samples were then visualized under an inverted florescence microscope (Olympus IX81) carrying out a malalignment of 4× images to show the whole well. Viability and toxicity caused by the laser application were also quantified by labeling cells with LIVE/DEAD kit as mentioned above and using a FACSAria BD cytometer (BD Bioscience). MSCs, B16-F1 cells, B16-F10 cells and monocytes without treatment were also assessed as control samples to obtain the basal viability/cytotoxicity status.

To study in more detail the photothermal applications, a co-culture of MSCs (previously loaded with PEG-HGNs) and B16-F1 was also irradiated to study the selective photothermal effect due to the specific nanoparticle tracking between cell lines. Therefore, MSCs and B16-F1 were co-cultured together onto a 24-well plate at a density of 2 × 10^4^ MSCs and 2 × 10^4^ B16-F1 per well. After 24 h, cells were irradiated as mentioned above. Finally, they were stained with LIVE/DEAD kit (Thermo Fisher Scientific, USA) and observed in an IX81 inverted fluorescence microscope (Olympus, Japan).

### Statistical analysis

The results here expressed as the mean ± standard deviation were carried out in triplicate. The software Stata/SE 12.0 was used to develop the statistical analyses of the data. This analysis was performed using Student’s t-test and one-way analysis of variance with a normal distribution whereas the Wilcoxon Rank-sum test and the Kruskal–Wallis test were used for the data groups with a non-normal distribution. P < 0.05 was considered statistically significant.

## Additional files

**Additional file 1.** Additional figures and tables.

**Additional file 2.** Time-lapse video.

**Additional file 3.** Time-lapse video.

## Abbreviations

NPs: nanoparticles
GNs: gold nanoparticles
HGNs: hollow gold nanoparticles
PEG-HGNs: PEGylated hollow gold nanoparticles
MSCs: human placental mesenchymal stem cells
