# Supplementary material for: Exosome origin determines cell targeting and the transfer of therapeutic nanoparticles towards target cells
Source: J Nanobiotechnology. 2019 Jan 25;17:16. doi: 10.1186/s12951-018-0437-z (PMC6346572; doi:10.1186/s12951-018-0437-z)
Supplement: Supplementary file 1 — Additional file 1. Additional figures and tables. [file 12951_2018_437_MOESM1_ESM.docx]

**Supporting Information**

Title: Exosome origin determines cell targeting and the transfer of therapeutic nanoparticles towards target cells

María Sancho-Albero, Nuria Navascués, Gracia Mendoza, Víctor Sebastián, Manuel Arruebo^*^, Pilar Martín-Duque^*^ and Jesús Santamaría

**Results**

HGNs uptake by MSCs

*Cytotoxicity test and cellular cycle assay*

As we would like to use the NPs for the tracking of the distribution of the exosomes cargo amongst cells and to verify whether they could be used as potential therapeutic vectors, we first tested the incorporation of the desired nanoparticles inside the cells. First of all, it was important to verify whether the PEG-HGNs were not affecting to the viability of the exosome-forming cells, therefore the viability of MSCs incubated with PEG-HGNs and HGNs was studied at concentrations from 0.015 to 0.5 mg mL−1 and compared to the control (untreated cells). The dose of 0.125 mg mL−1 was chosen as the limit for the subcytotoxic effect (**Figure S2**, in supporting information). Furthermore, we were concern that the inclusion of the nanoparticles inside the cells could affect growth patterns that might modify the exosome or microvesicle (such as apoptotic bodies) production. Then, the cell cycle was also analyzed before and after the treatment with HGNs and PEG-HGNs (**Figure S2**). It is possible to observe that the addition of both types of NPs to the MSCs cultures did not produce significant effects on cell cycle distribution at the dose studied (0.125 mg mL−1).

*Internalization of nanoparticles into MSCs*

Confocal microscopy was used to follow the internalization of HGNs and PEG-HGNs in MSCs (**Figure S2C**). Large amounts of PEG-HGNs (red) were observed by reflection inside MSCs cytosol forming aggregates > 200 nm. In comparison, less HGNs aggregates were visualized inside the cell cytoplasm. Several factors, such as physical or chemical properties as well as size and surface chemistry of nanoparticles influence their interaction with cell membranes and their intracellular uptake. We attribute the higher PEG-HGNs amounts within cells to their elevated stability and reduced agglomeration provided by the PEG coating in the culture media. A superior colloidal stability would enhance the cellular uptake whereas large aggregates cannot be phagocytized. As expected, both HGNs and PEG-HGNs were only distributed in the cytoplasm rather than in the nuclei, as evidenced by the orthogonal projections shown in **Figure S2C**. The amount of gold inside MSCs was indirectly quantified by elemental analysis of the supernatant after incubation of the cells with HGNs and PEG-HGNs at different times: 24, 48 and 72 h. Gold content was normalized by the number of cells present at each time point. In agreement with the confocal microscope images, it can be seen that significantly more NPs were internalized in MSCs at any time when they were coated with PEG (**Figure 2SD**). These results also reveal that PEG-HGNs and HGNs were present within MSCs for several days after their addition, although the highest gold content was observed 24h after nanoparticle incubation, reaching an average load of 1.12 ng of Au per cell. In contrast, only 0.08 ng of gold/cell was obtained inside MSCs after 24 h of incubation with HGNs. In the remainder of this work, a 24 h incubation period was used. Taking into account the particle dimensions and their physicochemical characteristics (see the materials and methods section), the particle uptake by MSCs after 24h incubation could be estimated at over 5 x 10^6^ nanoparticles for PEG-HGNs and 7 x 10^4^ for HGNs.

*Pathway of incorporation of PEG-HGNs into MSCs*

As the tracked nanoparticles would be delivered by exosomes, it was important to find out the preferential pathways to incorporate nanoparticles into the target cells and to verify if those mechanisms were compatible with the exosome processing inside the cells. We have used different types of chemical inhibitors including HCl (cytosol acidification), NaN3 (ATP depletion), sucrose (trapping clathrin in microcages), β-cyclodextrin (stopping caveolae-mediated endocytosis) and low temperature (inhibition of energy-dependent endocytosis) to identify the main NPs internalization pathway in MSCs. Our results show (**Figure S3**) that when energy-dependent pathways were inhibited (NaN3 and incubation at 4 ºC) a strong decrease in the internalization was observed. The same occurred when clathrin-dependent endocytosis was hindered by adding sucrose. On the other hand, when cells were treated with HCl, NPs were observed on the surroundings of the cell membrane, suggesting that HGNs and PEG-HGNs were trapped in clathrin pits. Also, the addition of β-cyclodextrin did not affect internalization, indicating that MSCs took nanoparticles up independently of the caveolae-mediated pathway. Finally, a study of HGNs co-localization with early and late endosomes was carried out, comparing cells cultured at 4 ºC or under normal physiological conditions. In agreement with the above results, the presence of HGNs and PEG-HGNs in cytosol was observed (**Figure S3**) only when the cells were grown in normal conditions and they co-localized with early endosomes, as could be expected after 5 h of NPs addition. In summary, the above results indicate that not only clathrin-mediated endocytosis, but also other energy-dependent pathways were the internalization mechanisms involved in PEG-HGNs capture by MSCs, and therefore nanoparticles could be efficiently incorporated in the target cells.

**Experimental section**

**Synthesis and characterization of HGNs**

Cobalt (II) chloride hexahydrate (CoCl_2_·6H_2_O), sodium citrate tribasic dehydrate (Na_3_C_6_H_5_O_7_·3H_2_O), poly (vinylpyrrolidone) (PVP) Mw=55000 Da, gold (III) chloride hydrate (50 % of purity), sodium borohydride (NaBH4), and poly (ethylene glycol)-ether thiol (SH-PEG, 1000 Da MW) were employed in the HGNs synthesis and were obtained from Sigma Aldrich. In brief, dH2O (400 mL), 0.4 M of Cobalt (II) chloride (400 μL) and 0.1 M sodium citrate (1.6 mL) were put in contact for 40 minutes in a two-necked round flask without magnetic stirring under an inert Ar atmosphere to avoid a premature Co oxidation. Then, 1 wt.% solution of PVP (2 mL) and 0.1 M NaBH_4_ (400 μL) were added. The color solution changed from pale pink to brown indicating the cobalt NPs production. During this step, the PVP served as a stabilizer and shape-controller of the NPs. Subsequently, dH_2_O (120 mL) and 0.1 M gold (III) chloride (180 μL) were mixed with 360 mL of the previous cobalt-based dispersion used as sacrificial template to initiate the formation of CoCl_2_ and the galvanic reduction of Au^3+^ obtaining hollow Au-based shells. The color change from brown to blue was indicative of the HGNs formation. Finally, the NPs dispersion was centrifuged at 10000 rpm for 10 min. Taking advantage of the strong chemical bond between Au and S, the resulting HGNs were coated with SH-PEG by putting them in contact during 1 h under magnetic stirring at room temperature. Any excess of unbound PEG was removed by dialysis against distilled water during 48 h.

**Cell culture conditions**

Human placental mesenchymal stem cells (MSCs) were obtained from Cellular Engineering Technologies (CET) (Coralville, IA, USA), B16-F1 and B16-F10, less and more metastatic murine skin melanoma cells respectively were provided by cell services from Cancer Research-UK, and monocytes were obtained from American Type Culture Collections (ATCC). MSCs were cultured in Dulbecco’s modified Eagle’s medium (DMEM; Biowest, France) supplemented with 5 μg mL^−1^ of FGF-2 growth factor (PeproTech, USA), with 10 % of fetal bovine serum (FBS, GIBCO, USA), 1 % penicillin/streptomycin and 1 % amphotericin (Biowest, France) and maintained at 37 ºC in a 5 % CO_2_-humidified atmosphere under hypoxic conditions (3% O_2_). For culturing B16-F1 and B16-F10 cells, DMEM with 10 % of FBS (GIBCO), supplemented with 1 % penicillin/streptomycin and 1 % amphotericin (Biowest, France) were used. Finally, monocytes were cultured in RMPI Medium 1640 (Biowest, France) supplemented with 10 % FBS (GIBCO), 1 % penicillin/streptomycin and 1 % amphotericin (Biowest, France). B16-F1 cells, B16-10 cells and monocytes were maintained under normoxic conditions. To obtain the culture media free of exosomes (Ultracen medium), they were depleted from serum by ultracentrifugation at 100000 g for 8 h at 4 ºC.

**Cellular uptake of NPs by MSCs**

*Confocal microscopy, flow cytometry and MP-AES*

The cellular uptake and the trafficking of HGNs and PEG-HGNs in MSCs were evaluated by confocal microscopy (Spectral Confocal Microscope Leica TCA SP2) with a 63x oil immersed N.A. 1.40 objective. Cells were seeded at a density of 2·10^4^ cells onto 20 mm cover slips (in a 24-well plate) and cultured for 24 h. The NPs (0.125 mg mL−1) resuspended in DMEM were added to the cells and incubated for 24 h. Afterwards, cells were fixed with para-formaldehyde (PFA) 4 %. In order to label the cytoplasmic actin, cells were stained with phalloidin-Alexa488 (Invitrogen, USA) and Draq-5 was used to observe the nuclei. Reflection of the incident light at 488/490 nm was used to directly visualized HGNs and PEG-HGNs-based agglomerates. Z-stack orthogonal projections were developed to determine the presence of NPs inside the cytosol.

A Gallios flow cytometer (Beckman Coulter) containing a photomultiplier tube side scatter (SSC) was used to evaluate whether the presence of both types of PEGylated and non-PEGylated NPs affected the cell granularity or the MSCs internal complexity. Briefly, 2.5 x 10^5^ cells per well were seeded onto a 6-well plate. Then, they were incubated with HGNs or PEG-HGNs (0.125 mg mL−1) for 24 h. Afterwards, MSCs were trypsinized and washed twice with PBS. Finally, cells were resuspended in PBS and measured by flow cytometry. The SSC percentage of MSCs treated with nanoparticles was compared to that obtained from the control sample (untreated cells).

Finally, to quantify the amount of gold inside MSCs, they were seeded onto 6-well plates at a density of 2.5 x 10^5^ cells per well and were grown for 24 h. Later, HGNs and PEG-HGNs dispersions (0.125 mg mL−1) were prepared freshly in DMEM and were added to the cell cultures for 24, 48 and 72 h. After these time points, cells were harvested and washed twice with PBS (1500 rpm, 5 min). Control samples (cells without treatment) were collected using the same protocol. Once the cellular pellets were obtained, they were digested with 10 % Aqua regia (HNO3 + 3HCl) in dH2O (1.5 mL). Digestion was performed at room temperature for 1 h. Total amount of gold derived from HGNs and PEG-HGNs was determined by MP-AES using a quadrupole ICP mass spectrometer (4100 MP-AES, Agilent Technologies, USA). Calibrations were carried out using Au standards in 10 % Aqua regia ranging from 0 to 10 ppm. Furthermore, considering nanoparticle volume as the volume of a sphere and knowing gold metal density, we could estimate the gold mass present in each particle (HGH and PEG-HGN). Thus, taking into account that all the cobalt present in the first steps of nanoparticle’s synthesis disappeared and knowing the thermogravimetric analysis (TGA) results of HGNs and PEG-HGNs recently published^30^, the number of NPs per cell at 24, 48 and 72 h was calculated.

*Identification of the cellular pathway*

To study the specific internalization mechanism of NPs in MSCs, different uptake pathways were blocked with a variety of chemical inhibitors previously reported^38,44^. The presence or absence of HGNs and PEG-HGNs in MSCs under the effect of the different inhibitors was detected by Z-stack orthogonal projections obtained from confocal microscopy (Spectral Confocal Microscope Leica TCA SP2) as mentioned above. In this case, cells were seeded at a density of 2 x 10^4^ cells onto 20 mm cover slips (in a 24-well plate) and allowed to grow for 24 h. Then, cells were pre-incubated with the different inhibitors for 1 h more. To block clathrin-dependent endocytosis, cells were put in contact with 500 mM sucrose to traps the clathrin in microcages (Sigma Aldrich, USA). To inhibit the scission of the clathrin pits from the membrane, cells were pre-incubated with 0.1 M HCl. For ATP depletion, cells were treated with 3 mg mL−1 NaN3 (Sigma Aldrich, USA). Disruption of caveolae-mediated endocytosis was assessed by adding 5 mM cyclodextrin (Sigma Aldrich, USA) as cholesterol depleting agent^44^. Finally, cells were also cultured at 4 ºC during 1 h to inhibit energy-dependent endocytosis. Once MSCs were treated with the mentioned chemical inhibitors, HGNs and PEG-HGNs (0.125 mg mL−1) were added to cell culture and incubated for 3 h.

The confocal microscopy co-localization experiments of nanoparticles inside early and late endosomes were carried out under the inhibition of the energy-dependent endocytosis (4 ºC) or under control conditions (cells cultured at 37 ºC). Here, 2 x 10^4^ MSCs were seeded onto 20 mm cover slips (in a 24-well plate). After 24 h of maintenance, HGNs and PEG-HGNs (0.125 mg mL^−1^) were added to the cell culture and were incubated during 5 h at 4 ºC or at 37 ºC, blocking energy-dependent endocytosis or leaving cells to growth under normal conditions respectively. Later, early and late endosomes were labeled with CellLight® Reagents BacMam 2.0 (Thermo Fisher Scientific, USA) following the established protocol. Finally, cells were fixed with 4 % PFA as mentioned above and were visualized in a confocal microscope (Spectral Confocal Microscope Leica TCA SP2). Again, nanoparticle-based agglomerates were observed directly by reflection and nuclei were labeled with Draq-5.

**Exosome isolation and characterization**

MSCs-EXOs were isolated following a protocol based on successive ultracentrifugation cycles from cell culture supernatants of MSCs. Cells were cultured until confluence. To remove remaining debris, supernatants were centrifuged for 20 min at 2000 g and at 4 ºC. For the elimination of the microvesicles, another centrifugation step was carried out for 1 h at 10000 g and at 4 ºC. To obtain the exosomes fraction, the samples were ultracentrifuged twice for 2 h at 100000 g and at 4 ºC. The obtained precipitates were resuspended in PBS and a Pierce BCA protein assay (Thermo Fisher Scientific, USA) was performed in order to estimate the protein content in the exosomal sample. MSCs-EXOs were characterized by TEM. To visualize exosomes, phosphotungstic acid (3 %) was used as a negative contrast agent. From TEM images, the average diameter of MSCs-EXOs was obtained using ImageJ software (NIH-RSB). Exosome zeta potential (surface charge) was determined at pH=7 in PBS (Brookhaven 90 plus and ZetaPALS software). To identify specific surface proteins such as CD9 and CD63, western blotting was carried out. Briefly, 25 μg of MSCs-EXOs were precipitated with acetone (1:1 w/w), lysated in Laemmli buffer (Sigma Aldrich, USA) and boiled at 95 ºC during 5 min. Proteins were then separated by 12 % SDS-polyacrylamide gel electrophoresis during 2 h and transferred to nitrocellulose membranes during 4 h at 4ºC. The membranes were blocked overnight with non-fat dry milk in tris-buffered saline (TBS) 5 %. After that, blots were incubated with primary antibodies GAPDH, 1:1000, CD9; 1:2000 (Abcam UK) and CD63, 1:1000 (BD Biosciences, United States). Membranes were washed three times with TBS-Tween (TBST) followed by the incubation with the secondary antibody (Sigma-Aldrich, USA). Finally, membranes were extensively washed and chemiluminescence substrate was added and imaging was carried out. Western-Blot experiments were assessed considering the number of cells (exosomes derived from 10^6^ cells were measured when they were untreated or treated with nanoparticles) in order to compare whether the presence of PEG-HGNs affect the exosome secretion. The concentration and size distribution of exosomes were measured employing Nanosight (Malvern Instruments, UK). Samples were diluted in PBS before the measurements in order to optimize the number of particles in the field of view. Samples were measured at room temperature in triplicate for 60 seconds.

**Gold nanoparticles-loaded exosomes**

The presence of PEG-HGNs in exosomes was evaluated by confocal microscopy and by TEM. For the confocal studies, cells were seeded at a density of 2 x 10^4^ cells onto 20 mm cover slips (in a 24-well plate) and incubated with PEG-HGNs (0.125 mg mL^−1^). Afterwards, cells were fixed with 4 % PFA and an immunocytochemistry was performed: to label the cytoplasmic actin, cells were marked with phalloidin-Alexa543 (Invitrogen), for exosomes staining, CD63-Alexa488 antibody (Thermo Fisher Scientific, USA) was employed and nuclei were stained with Draq-5. Nanoparticle-based agglomerates were again directly visualized by reflection of the incident light in the microscope at 488/490 nm ex/em. Z-stack orthogonal projections were developed to determine the presence of NPs inside MSCs-EXOs. For electron microscopy, isolated exosomes were deposited on Formvar carbon-coated grids to be further stained and visualized as mentioned above. Protein expression (CD9 and CD63) was evaluated by Western Blot analysis charging exosomes secreted by the same number of cells in order to be able to compare the protein expression when cells were treated or not with nanoparticles. A Pierce BCA protein assay was also performed as mentioned above in order to estimate the protein content in the exosomes sample secreted by 10^6^ MSCs when cells were treated or not with nanoparticles. Exosome zeta potential (surface charge) was also measured as previously mentioned. NTA measurements were also performed to obtain the size distribution and the concentration of PEG-HGNs_MSCs_EXOs.

**Exosomes as selective vectors of PEG-HGNs uptake between different cell lines**

*Optimization of co-culture conditions by flow cytometry and fluorescence microscopy*

For the evaluation of PEG-HGNs transference through exosomes between monocytes and MSCs, the cell lines were cultured under a variety of conditions, including separately culture of MSCs and monocytes, and a simultaneous co-culture of both cell lines. In order to optimize the co-culture conditions, specific cell surface markers of monocytes and MSCs (CD19, CD14, CD34, CD45, CD73, CD90, CD10 and, HLA-DR) were evaluated by flow cytometry (FACSAria BD cytometer, BD Bioscience) when cells were cultured separately or together. For that, cells were seeded onto 6-well plates at a density of 2.5 x 10^5^ cells per well under normoxic conditions. In the case of co-cultured conditions 1.25 x 10^5^ monocytes and 1.25 x 10^5^ MSCs were seeded in the same well. After 24 h of maintenance, PEG-HGNs (0.125 mg mL−1) were added for 24 and 48 h. Then, cells were trypsinized and washed twice with PBS (1200 rpm, 5 m) being analyzed by flow cytometry. Monocytes and MSCs were also labeled with PKH67 and PKH26 linkers (Sigma Aldrich, USA) respectively and samples were visualized using an IX81 inverted fluorescence microscope with 10x and 20x objectives (Olympus, Japan).

**Figure captions:**

**Figure S1.** Stability evaluation of HGNs and PEG-HGNs in culture media. A) TEM images of both NPs dispersions incubated for 24 h in culture media. B) BCA assay of HGNs and PEG-HGNs suspended in DMEM for 72 h. C) Surface charge analysis of naked and PEGylated NPs.

**Figure S2**. Cytocompatibility of NPs on MSCs. A) Alamar Blue assay showing the cytotoxicity of HGNs and PEG-HGNs at different time points (24 h, 48 h and 72 h). B) Distribution of cell cycle phases in MSCs assayed after treatment with NPs for 48 h. C) Gold content analysis evaluated by MP-AES inside cells present in the different time points and incubated at the sub-cytotoxic dose. The amount of gold and the number of NPs were normalized by the number of cells for each time point. Significant differences (p < 0.05) with PEG-HGNs at 24 h were observed. D) HGNs and PEG-HGNs internalization evaluation by confocal microscopy. NPs aggregates were directly visualized by reflection inside cells (red channel). DIC stands for differential interference contrast.

**Figure S3**. Endocytosis mechanisms for HGNs and PEG-HGNs on MSCs. A) Confocal images showed NPs uptake in the presence of HCl, NaN3, sucrose, β cyclodextrin or when cells were cultured at 4 ºC. ATP-dependent and energy-mediated endocytosis where the main internalization mechanisms. B) Early and late endosomes co-localization study with NPs when endocytosis was inhibited at 4 º or under normal conditions.

**Figure S4**. Co-culture of MSCs and THP1 cell lines observed by inverted florescence microscopy. MSCs and monocytes were labelled with PKH67 and PKH26 linkers, respectively. B) Evaluation of specific cell surface markers of these both cell lines when cells were cultured separately or under co-culture conditions (CD19, CD14, CD34, CD45, CD73, CD90, CD105 and HLA-DR).

**Figure S5**. A) MP-AES assay to quantify PEG-HGNs loaded exosomes distribution (gold content) between monocytes and MSCs when they were co-cultured simultaneously with PEG-HGNs_MSCs-EXOs or with PEG-HGNs_Monocytes-EXOs during 24 h or 48 hours. Sn: stands for supernatant.

**Figure S6**. Live/dead images obtained for MSCs, B16-F1 and B16-F10 and monocytes when they were treated with MSC-derived exosomes loaded with PEG-HGNs.

**Scheme 1**. A simplified description of the processes involved in the processing of PEG-HGNs by MSCs.
